# Mesenchymal stem cell-derived exosomes: a promising alternative in the therapy of preeclampsia

**DOI:** 10.1186/s13287-024-03652-0

**Published:** 2024-02-05

**Authors:** Haoran Shi, Zejun Yang, Jianjian Cui, Hui Tao, Ruilin Ma, Yin Zhao

**Affiliations:** 1 Department of Obstetrics and Gynecology, Union Hospital, Tongji Medical College, Huazhong University of Science and Technology, No. 1277 Jiefang Avenue, Wuhan, 430022 China; 2 Shenzhen Huazhong University of Science and Technology Research Institute, Shen Zhen, 518000 China

**Keywords:** Angiogenesis, Exosomes, Engineered exosomes, Immune regulation, Mesenchymal stem cells, Oxidative stress, Preeclampsia

## Abstract

Preeclampsia (PE) is a common morbid complication during pregnancy, affecting 2%-8% of pregnancies globally and posing serous risks to the health of both mother and fetus. Currently, the only effective treatment for PE is timely termination of pregnancy, which comes with increased perinatal risks. However, there is no effective way to delay pathological progress and improve maternal and fetal outcomes. In light of this, it is of great significance to seek effective therapeutic strategies for PE. Exosomes which are nanoparticles carrying bioactive substances such as proteins, lipids, and nucleic acids, have emerged as a novel vehicle for intercellular communication. Mesenchymal stem cell-derived exosomes (MSC-Exos) participate in various important physiological processes, including immune regulation, cell proliferation and migration, and angiogenesis, and have shown promising potential in tissue repair and disease treatment. Recently, MSC-Exos therapy has gained popularity in the treatment of ischaemic diseases, immune dysfunction, inflammatory diseases, and other fields due to their minimal immunogenicity, characteristics similar to donor cells, ease of storage, and low risk of tumor formation. This review elaborates on the potential therapeutic mechanism of MSC-Exos in treating preeclampsia, considering the main pathogenic factors of the condition, including placental vascular dysplasia, immunological disorders, and oxidative stress, based on the biological function of MSC-Exos. Additionally, we discuss in depth the advantages and challenges of MSC-Exos as a novel acellular therapeutic agent in preeclampsia treatment.

## Background

Preeclampsia (PE), characterized by new-onset hypertension after 20 weeks of gestation, is a common pregnancy complication concomitant with multisystem functional obstacles such as elevated liver enzymes, thrombocytopenia, proteinuria, renal insufficiency, persistent severe headache, and seizures [1]. It is becoming increasingly common in the developed countries and remains a major cause of maternal and fetal morbidity and mortality in the developing countries [2]. The pathogenesis of PE is complex, involving dysfunctions of trophoblasts, deficient spiral arterial remodeling, maldevelopment of the placental vasculature leading to maternal–fetal perfusion deficiency, oxidative stress, imbalance of maternal–fetal immune regulation [3]. The only effective treatment for PE is pregnancy termination, but it comes with risks such as fetal growth restriction and preterm delivery. Moreover, pregnant women have an increased risk of cardiovascular and kidney diseases after delivery [3]. Therefore, identifying a treatment that can effectively delay the pathological progression of PE is important for improving maternal and neonatal outcomes. Mesenchymal stem cell-derived exosomes (MSC-Exos) exert a vital regulatory influence on endothelial cell [4] and trophoblast [5] function, immune response [6], and antioxidant stress [7]. They hold great promise as an alternative therapeutic option to MSCs, offering a novel therapeutic avenue for the treatment of PE.

MSCs possess multidirectional differentiation potential, and exhibit advantages such as rapid self-renewal, stable doubling time, and high proliferation capacity [8]. MSCs attenuate the pathological progression of PE and enhance outcomes for both mother and infant [9, 10]. Initially, MSCs were believed to rely on homing and differentiation for their therapeutic effects; however, subsequent studies have underscored the role of paracrine action [11]. As one of the main paracrine pathways of MSCs, exosomes transport a diverse range of bioactive substances secreted by MSCs and serve various transport functions. MSC-Exos offer advantages over MSCs, including enhanced biological stability, reduced immunogenicity, lower lung interception, and the ability to traverse the placental barrier [12, 13]. Moreover, they mitigate potential risks associated with chromosomal variations, tumourigenicity, thrombosis, and immune rejection that may arise during MSCs therapy [14]. MSC-Exos have progressively emerged as a focal point of research in numerous fields, encompassing inflammation [15], autoimmunity [16], ischaemia [17], cerebrovascular, and neurodegenerative disorders [18]. Preclinical data indicate that treating disorders with MSC-Exos may offer greater safer and more versatility compared to MSC-base therapies [19].

## Exosomes of mesenchymal stem cells

Exosomes, initially discovered in sheep reticulocytes in 1983, originate from the nuclear endosome lysosome system [20]. Their production involves intricate molecular mechanisms encompassing endocytosis, content sorting, and trafficking. The key molecules involved in this process include ceramide [21], tetra-spanning membrane proteins [22, 23], and the endosomal sorting complex required for transport [24]. Exosomes are extracellular vesicles with a bilayered lipid membrane structure similar to that of the plasma membrane. With a diameter of 40–100 nm and a density of 1.13–1.19 g/mL, these vesicles contain proteins, mRNA, miRNA, DNA, lipids, cytokines, transcription factor receptors, and other genetic material. Exosomes proficiently transfer bioactive substances, which are prone to inactivation or degradation through various pathways, to target cells, modulating their biochemical characteristics and participating in regulatory processes such as tissue repair, tumour diagnosis and treatment, and immunomodulation [20, 25]. The interaction between exosomes and target cells encompasses three main pathways: direct activation of target cell membrane receptors, modification of the extracellular environment surrounding target cells, fusion with the cell membrane, and release of bioactive molecules into the target cells [26]. Under transmission electron microscopy, exosomes exhibit a distinctive cup-shaped appearance, while they appear as isolated spheres under low-temperature electron microscopy [27]. Compared to synthetic vectors like liposomes and nanoparticles, exosomes possess unique advantages in disease diagnosis and treatment due to their inherent nature and heterogeneity [20]. They manifest diverse functions, including promoting cell proliferation and migration, regulating immune and anti-inflammatory responses, and are widely used in disease repair, including facilitating endometrial repair in uterine adhesion disease [28], ameliorating symptoms of ovarian insufficiency and polycystic ovary syndrome [29], treating PE [30, 31], repairing spinal cord injury [32], and expediting cutaneous wound healing in diabetic mice [33, 34].

Exosomes exist in multiple body fluids such as blood, breast milk, semen, and saliva [35]. Almost all types of normal cells, including human umbilical vein endothelial cells (HUVECs), MSCs, T cells, B cells, macrophages, dendritic cells, and natural killer cells, produce exosomes. Among these cells, MSCs are multipotent stem cells with self-renewing and multidirectional differentiation capabilities. They exhibit a strong paracrine activity and secret many exosomes [20]. MSCs can be exacted from various sources, such as placenta, umbilical cord blood, amniotic fluid, adipose tissue, bone marrow, and even brain tissue[36]. Compared to other types of stem cells (embryonic stem cells and induced pluripotent stem cells), MSCs have several advantages: 1) relatively easy extraction from various tissues such as bone marrow, peripheral blood, and adipose tissue, 2) low cost of isolation and culture, 3) immunosuppressive ability, 4) versatile treatments for patients with allotransplantation and autologous transplantation [37], and 5) low immunogenicity owing to the lack of MHC-II and low expression of MHC-I, similar to their parental cells [16]. Since 2010, over 100 registered clinical trials have been conducted to evaluate the effectiveness of MSCs in treating various diseases [38], such as osteoarthritis [21], COVID-19 [39], cerebral palsy [40], and heart failure [41].

## Therapeutic potential of MSC-Exos in PE

MSC-Exos have the potential to therapeutically to delay the progression of PE and enhance outcomes by enhancing trophoblast function [31], promoting placental angiogenesis [42], regulating immune responses [6], reducing inflammatory [43] and oxidative stress [7] (Table 1).

**Table 1:** Therapeutic theory of MSC-exos in enhancing trophoblast function, promoting angiogenesis, regulating immune responses and reducing oxidative stress

Source of exosome	Therapeutic theory	Molecular target	References
BM-MSCs	AKT signaling pathway activation PI3K/AKT signaling pathway activation↑VEGF,Ang-1 ↑IL-10,TGF-β ↓IL-1β,IL-6,TNF-α,IL-12	↑H19, FOXO1↓let-7b PIK3R2 inhibition mediated by exosomal miR-126 Not reported	[47] [54] [63, 64]
hUC-MSCs	ERK/MMP2 signaling pathway activation TIM3/mTORc signaling pathway deactivation Not reported Not reported ERK1/2 and AKT signaling pathway activation β-catenin signaling pathway activation ↓ROS, ↓NOX1,NOX4	tyrosine phosphatase inhibition mediated by exosomal miR-139-5p Notch2 inhibition mediated by exosomal miR-18b BRD4 inhibition mediated by exosomal miR-101 FSTL3 inhibition mediated by exosomal miR-140-5p SPRED-1 and PIK3R2 inhibition mediated by exosomal miR-126-3p Wnt4 Not reported Not reported	[30] [48] [49] [50] [56] [57] [66, 67] [68]
AF-MSCs	EZH2/mTOR signaling pathway deactivation	Not reported	[5]
AD-MSCs	Not reported ↑tolDC	Delta-like ligand 4 inhibition mediated by exosomal miR-125a Not reported	[53] [61]
PMSCs	Not reported	ICAM-1 promotion mediated by exosomal miR-130b-3p	[55]
DMSCs	↓malondialdehyde,IL-6	Not reported	[69]

↓and↑show the decrease and the increase, respectively

BM-MSCs:bone marrow mesenchymal stem cells, hUC-MSCs:human umbilical cord mesenchymal stem cells, AF-MSCs:amniotic fluid mesenchymal stem cells, AD-MSCs:adipose tissue mesenchymal stem cells, PMSCs:placenta mesenchymal stem cells, DMSCs:decidual mesenchymal stem cells

## MSC-Exos promote trophoblast proliferation, migration, and invasion

The placenta is crucial for the exchange of gases, nutrients, and metabolites between mother and fetus. Proper development of placental vessels are the premise and basis for the smooth progression of pregnancy and adequate blood perfusion to the fetus. PE is characterized by impaired placentation, abnormal function of extravillous trophoblast cell, and compromised uterine spiral artery remodeling during placental development [44–46].

**Fig. 1:**
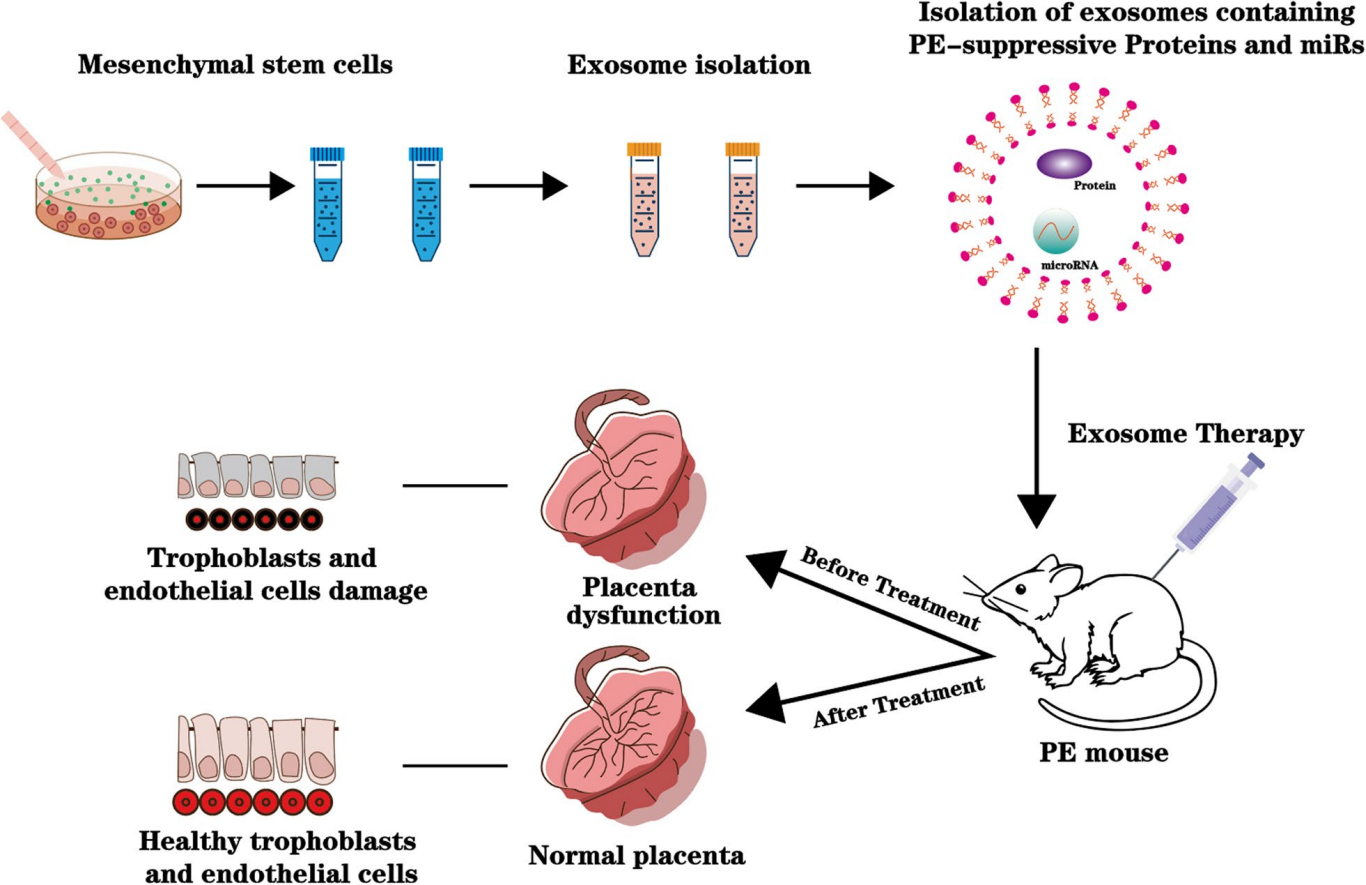
A schematic view of the exosome therapy for PE treatment using MSC-derived exosomes containing PE-suppressive proteins and miRs

MSC-Exos have been shown to enhance the biological functions of trophoblasts. For instance, patients with PE have higher expression of let-7b and lower expression of FOXO1 in the placental tissues compared to control. H19 acting as a competitive RNA for let-7b directly targets FOXO1. MSCs-Exos can transport H19 to trophoblasts, resulting in reduced let-7b expression, increased FOXO1 expression, activation the AKT signaling pathway, and ultimately enhancing the invasion and migration of trophoblasts and inhibiting their apoptosis. This study offers new insights into PE treatment [47]. In another study, exosomal miR-139-5p from human umbilical cord mesenchymal stem cells (hUCMSC-Exos) has been demonstrated to accelerate trophoblast invasion and migration, inhibited trophoblast apoptosis by downregulating protein tyrosine phosphatase expression, and activated the ERK/MMP-2 pathway, thereby improving PE symptoms in rats [30]. Additionally, a study discovered that the expression of miR-18b was downregulated while Notch2, TIM3, and mTORC1 levels were increased in the placenta of patients with PE. hUCMSC-Exos promoted trophoblast migration by secreting microRNA-18b to inhibit Notch2 expression in trophoblasts. They further applied hUCMSC-Exos to a rat model of PE and found that they improved the symptoms of PE in pregnant rats [48] (Fig. 1). Furthermore, hUCMSC-Exos were found to promote trophoblast migration and invasion by transferring miR-101 to trophoblasts and inhibiting BRD4 expression. In addition to promoting trophoblast migration and invasion, MSC-Exos promoted autophagy and trophoblast proliferation under hypoxic conditions [49]. Jiang et al. discovered that hUCMSC-Exos inhibited FSTL3 expression by transmitting miR140-5p, and further suppressed trophoblast inflammation under hypoxic conditions and promoted proliferation, migration, and invasion of hypoxic trophoblasts [50]. Exosomes derived from amniotic MScs enhance autophagy in trophoblasts by inhibiting the EZH2/mTOR signalling pathway, thus promoting their proliferation [5].

In summary, MSC-Exos promote the proliferation, migration, and invasion of trophoblasts and improve spiral artery remodeling, offering a new avenue for research on PE treatment.

### Mesenchymal stem cell exosomes promote angiogenesis by regulating endothelial cell function

Endothelial cells maintain vascular integrity, regulate thrombosis, and transport and perform barrier functions. They are an essential component of placental blood vessels and crucial for the proper functioning of the placenta. In patients with preeclampsia, placental dysfunction is often accompanied by impaired HUVEC function [20]. Therefore, targeted modulation of HUVEC function may be an effective therapeutic option for treating PE.

It has been found that hUCMSC-Exos can restore soluble fms-like tyrosine kinase-1(sFlt-1)-induced endothelial dysfunction in preeclampsia, thereby improving adverse pregnancy outcomes in PE mice [51]. Zhu et al. demonstrated that adipose tissue mesenchymal stem cell exosomes (ADMSC-Exos) accelerate the proliferation of HUVEC in a concentration-dependent manner [52]. In addition to the aforementioned effects, ADMSC-Exos transfer microRNA-125a to HUVEC, directly inhibiting its downstream target delta-like ligand 4 and promoting endothelial tip cell formation and angiogenesis [53]. Bone marrow-derived exosomes (BMMSC-Exos) upregulate vascular endothelial growth factor (VEGF) and human angiopoietin-1 (Ang-1) expression in HUVECs via microRNA-126. They also activate the PI3K/AKT signaling pathway by targeting PIK3R2, thereby promoting angiogenesis [54]. Gao et al. conducted observations revealing that after MSC-Exos derived from the placental tissues of patients with gestational diabetes with knockdown of microRNA-130b-3p upregulated ICAM-1 expression, leading to enhanced proliferation, migration, and angiogenesis ability of HUVECs [55]. Qu et al. revealed that hUCMSC-Exos overexpressing miR-126-3p downregulated the expression of SPRED-1 and PIK3R2, activating the ERK1/2 and AKT signaling pathways, and promoting the proliferation, migration, and angiogenesis of HUVECs [56]. Additionally, proteins carried by MSC-Exos play crucial roles in endothelial cells. As an illustration, hUCMSC-Exos activated β-catenin signaling pathway in endothelial cells through the Wnt4 protein, thereby promoting proliferation, migration, and angiogenesis of endothelial cells. Furthermore, in a rat skin-burn model, hUCMSC-Exos accelerated wound healing in vivo by promoting angiogenesis [57].

**Fig. 2:**
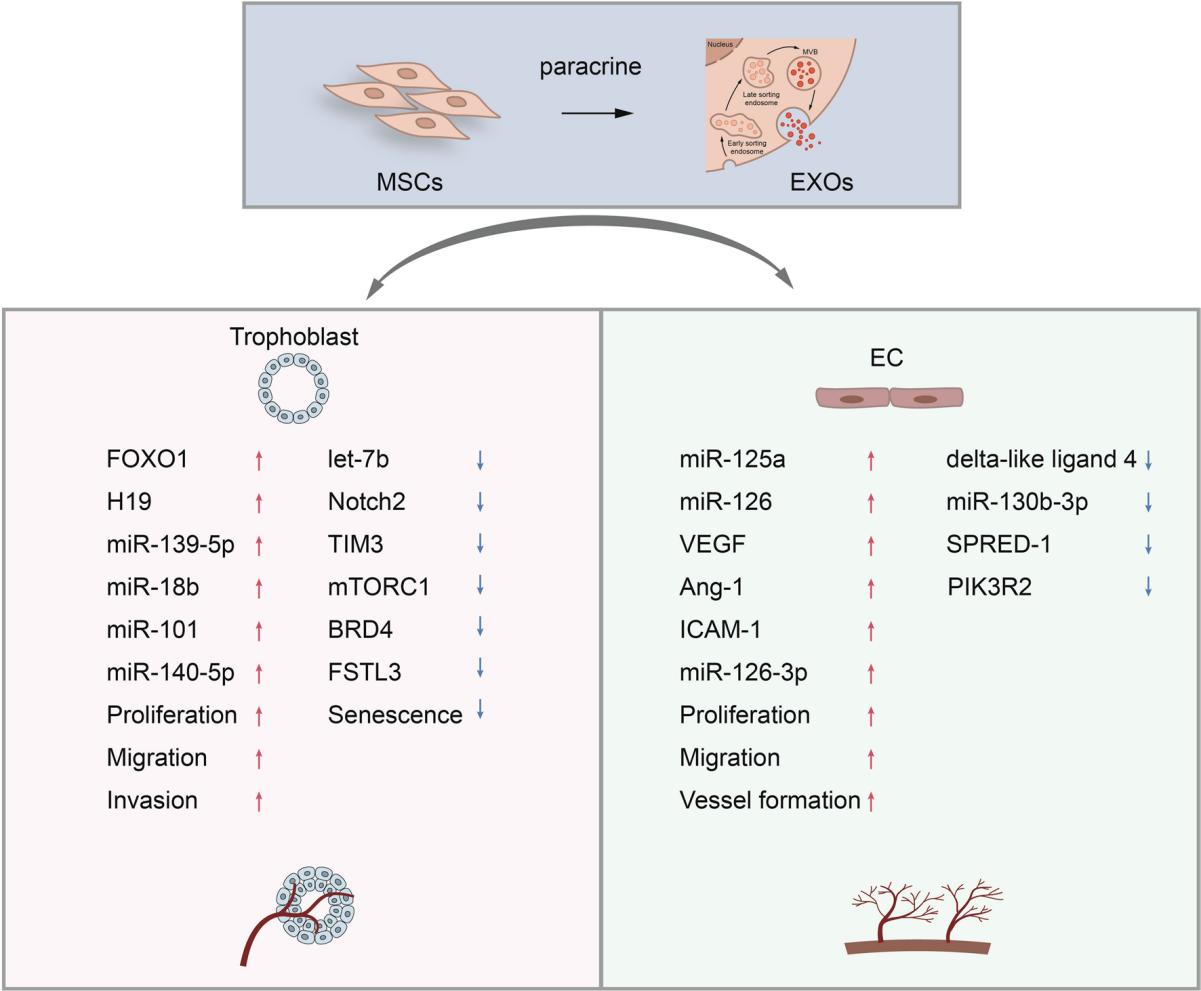
Mesenchymal stem cell exosomes promote trophblast and endothelial cell functions by upregulating or downregulating proteins and miRs

Overall, MSC-Exos enhance the proliferation, migration, and angiogenesis of endothelial cells through diverse molecular mechanisms and have the potential to promote placental angiogenesis and improve placental blood perfusion (Fig. 2).

### Immunomodulatory and anti-inflammatory effects of exosomes of mesenchymal stem cells

Immune regulation is crucial for ensuring a safe pregnancy. In solid organ transplantation, the recipient’s immune system recognizes alloantigens expressed by grafts, leading to immune attacks and rejection of transplanted organs, which can only be prevented by therapeutic immunosuppression. Similarly, during pregnancy, the maternal immune system may recognize antigens expressed by the fetus from father, potentially leading to rejection. The maternal immune tolerance mechanism suppresses such immune rejection. Therefore, maintaining normal maternal immune tolerance is essential for a successful pregnancy. Disruption of normal immune tolerance leads to pathological conditions such as PE, which has been associated with immune imbalance at the maternal–foetal interface [58]. In addition, placental ischaemia and hypoxia can cause an increase in reactive oxygen species (ROS) and ATP deficiency, ultimately promoting excessive production of inflammatory mediators, and contributing to PE [59].

MSC-Exos have emerged as a potential treatment for PE by regulating the uterine immune microenvironment. Taglauer et al. utilized a mouse model with preeclampsia-like features and administered hUCMSC-Exos during early pregnancy, revealing that hUCMSC-Exos promote the local recruitment of NK cells and macrophages in the uterus, as well as the expression of immune factors such as interleukin-10 (IL-10), interferon-gamma, and tumour necrosis factor-α (TNF-α). By regulating the immune microenvironment at the maternal–fetal interface, pregnancy outcomes were improved [60]. Additionally, MSC-Exos can exert anti-inflammatory effects by modulating the phenotype of inflammatory immune cells, such as inducing dendritic cells (DCs) and M1 macrophages to transform into tolerant dendritic cells (tolDCs) [61] and M2 macrophages [62], respectively. Studies have shown that ADMSC-Exos could induce mouse bone marrow-derived DCs to become tolDCs, thereby suppressing the immune response [61]. Furthermore, miRNAs enriched in MSC-exos are associated with macrophage immunoregulation and their immunomodulatory function can promote endometrial regeneration and fertility recovery [62]. In vitro and in vivo study have demonstrated that MSC-Exos inhibited the expression of pro-inflammatory cytokines such as IL-1β, IL-6, IL-12 and TNF-α, while increasing the expression of anti-inflammatory cytokines such as IL-10 and TGF-β [63, 64].

**Fig. 3:**
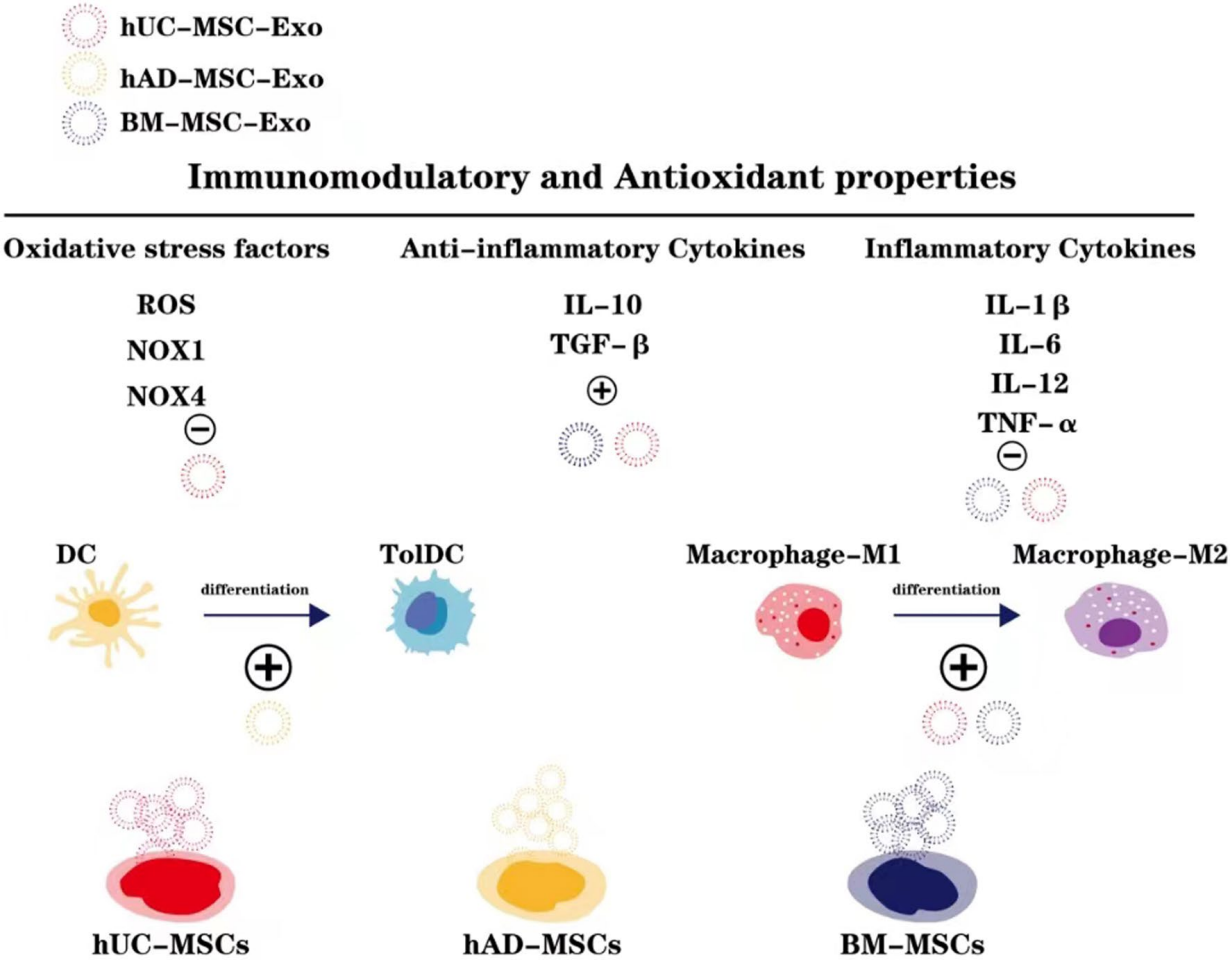
The possible modulatory effect of MSC-Exos on the dysregulated immune system components and oxidative stress in the experiments. The expression of oxidative stress factors can be reduced by hUC-MSC-Exos. BM-MSC-Exos and hUC-MSC-Exos can reduce over-expressed proinfammatory cytokines and increase the expression of anti-inflammatory cytokines. hAD-MSC-Exos can induce dendritic cells (DC) to transform into tolerant dendritic cells (tolDC) hUC-MSC-Exos and BM-MSC-Exos can induce M1 macrophages to transform into M2 macrophages

Hence, MSC-Exos play a crucial role in immune modulation and anti-inflammatory effects, presenting vast prospects for their application in the treatment of preeclampsia (Fig. 3).

### Antioxidant properties of mesenchymal stem cell exosomes

Oxidative stress plays a crucial role in the pathophysiology of PE [3]. Insufficient placental vascular perfusion resulting in ischaemia and hypoxia can lead to an increased release of ROS and reactive nitrogen species and decreased secretion of antioxidant factors. This imbalance between pro-oxidative and antioxidant capacities induces oxidative stress-related damage to proteins, lipids, and DNA, ultimately contributing to pregnancy complications such as PE [65]. Therefore, effective treatments that inhibit the release of oxidative stress factors and control oxidative stress response should be identified.

Several studies have indicated that MSC-Exos can effectively reduce oxidative stress levels, and alleviate tissue ischemia–reperfusion injury, and inhibit ROS-induced cell apoptosis [66, 67]. Under high glucose-induced oxidative stress conditions, hUCMSC-exos demonstrated concentration-dependent reduction in the expression of the oxidative stress factors NOX1 and NOX4 in HUVECs, indicating their ability to ameliorate oxidative stress damage [68]. Extracellular vesicles secreted by human decidual MSCs significantly reduced malondialdehyde level(a by-product of lipid peroxidation that causes systemic endothelial dysfunction in pregnant women with PE) and suppressed IL-6 expression in HUVECs treated with PE serum. This inhibition of oxidative stress and inflammation, consequently enhanced the proliferation and angiogenesis of HUVECs [69]. These findings suggest that MSC-Exos may have therapeutic potential for addressing oxidative stress in PE.

### Experimental research on enhancing pregnancy outcomes using mesenchymal stem cells-derived exosomes

PE is a serious pregnancy complication, and preterm delivery or miscarriage may occur during disease progression. MSC-Exos effectively reduced premature delivery and abortion rates. MSC derived from human amniotic fluid (AF-MSC) have been shown to inhibit inflammatory responses in trophoblasts induced by lipopolysaccharides (LPS) through the paracrine pathway. AF-MSC-Exos, which are rich in miR-146a-5p, miR-548e-5p, may serve as molecular mediators in reducing the LPS-induced trophoblast inflammation [59]. In rat models of abortion, the injection of BMMSC-Exos into the uterine horns resulted in significantly lower expression levels of IL-12, TNF-α, and IFN- γ at the maternal-foetal interface while the expression levels of IL-4 and IL-10 were adverse. This suggests that MSC-Exos can regulate the uterine immune response by inhibiting the Th1-type immune response and promoting the Th2-type immune response, thereby reducing the abortion rate [70]. These findings offer new hope for improving PE pregnancy outcomes.

## Enhancing the therapeutic effect of MSC-Exos

The composition of biomolecules in exosomes varies depending on the microenvironment and source cell conditions. Exosome therapy shows promise as a method for treating PE due to its advantages, including lower immunogenicity, enhanced biological stability, controllable graft doses, and efficient targeted delivery to cells. However, despite a higher homing rate compared to MSC therapy, most intravenously infused exosomes become trapped in the liver by the mononuclear phagocytic system, preventing them from reaching the target site of injury [71]. To overcome this limitation and maximize therapeutic effects, various optimization methods have been explored, such as genetic modification of MSCs to release functional exosomes, direct modification of exosomes with homing peptides to enhance targeted homing ability, pretreatment of MSCs with bioactive molecules, or modification of MSC culture conditions to enhance the therapeutic potential of MSC-Exos.

Multiple studies have demonstrated the therapeutic effects of hypoxic preconditioned MSCs in various animal disease models [72–74]. Hypoxic preconditioning of MSCs can elevate the levels of paracrine factors in MSC-Exos, thereby enhancing their reparative actions [75–79]. This includes increased upregulation of pro-angiogenic proteins like VEGF, epidermal growth factor, fibr0blast growth factor, and their receptors [77], promoting proliferation, migration, and tubular ability of HUVECs.

Genetic engineering is also a valuable approach to enhance the therapeutic effects of MSC-Exos [80]. Lentivirus-infected MSCs carrying an overexpression vector of HIF-1α produce exosomes that upregulate the expression of pro-angiogenic factors (VEGF, Ang-1, and PDGF) in hypoxia-treated HUVECs, effectively reversing the impaired migration, proliferation, and pro-angiogenic ability caused by hypoxia treatment [81]. These findings highlight the efficacy of genetic modification in enhancing the functional properties of MSC-Exos, particularly in promoting the function of endothelial cells and trophoblasts.

Despite the promising potential of exosomes in therapeutic applications, their targeting ability in animal experiments is limited, leading to challenges such as short half-life and reduced therapeutic efficacy. Biochemical engineering offers a simpler, faster, and more effective approach by directly modifying exosomes without the need for cell manipulation, thereby enhancing specific exosome secretion [18]. In a study, a central nervous system-specific rabies virus glycoprotein (RVG) peptide was conjugated to MSC-Exos surfaces using G-protein coupling, which outperformed unmodified exosomes in enhancing cognitive function in APP/PS1 mouse model [82]. Another example is the use of a tumor-homing peptide iRGD, which selectively binds to the placental surface in humans and mice without interfering with normal development. Hence, iRDG-exosomes may contain vital proteins or genes that specifically target the placenta and play crucial roles in PE treatment [83].

Considering the potential impact of multiple chemical reactions involved in biochemical engineering on exosome function, it is crucial to explore less invasive methods for modifying exosomes and enhancing their targeting abilities. One promising strategy is to load exogenous substances into the interior of exosomes instead of performing surface modifications [18]. In a study, iron oxide nanoparticles (IONP) were incubated with MSCs to generate exosome-mimetic nanovesicles (NV-IONP). These NV-IONPs can be magnetically guided to target injured spinal cord tissue with the aid of an external magnetic field (MF). Furthermore, the IONPs slowly release iron ions to activate the JNK and c-Jun signaling cascade in MSCs, enabling NV-IONPs to carry more therapeutic growth factors and improve the proliferation and migration capabilities of HUVECs [84].

MSCs loaded with specific drugs can optimize the composition of their secreted exosomes, leading to improved therapeutic outcomes. Various compounds such as atorvastatin (ATV) [85], pioglitazone (PGZ) [86], baicalin [87], oxytocin [88], curcumin [89], and hemin [90] have been explored to enhance MSCs’ survival rates and functions. For instance, exosomes derived from ATV-pretreated MSCs enhanced HUVECs’ migration and angiogenesis via the upregration of lncRNAH19 expression [91].

## Discussion

PE can result in adverse maternal and fetal pregnancy outcomes, which are common pregnancy complications [3]. However, the treatment options for PE are limited, often leading to pregnancy termination due to ineffective control of disease progression [92]. MSC-Exos carry numerous proteins, nucleic acids, and lipids that can be transported across cell membranes to modify signal transduction and gene expression in the target cells [33], thereby regulating the biological function of the target cells and promoting angiogenesis by improving the function of trophoblasts and endothelial cells. Furthermore, MSC-Exos play crucial roles in immune response regulation[6], anti-inflammatory processes [43], and antioxidant stress [7]. In conclusion, MSC-Exos have the potential to effectively treat PE by regulating multiple aspects of its pathogenesis.

Owing to potential chromosomal variations, tumourigenicity, thrombosis, immune rejection, and other challenges, progress in using MSCs for PE treatment has limitations. Exosomes, the crucial paracrine product of MSCs, serve as carriers to transport bioactive components to the surrounding cells and the circulatory system, even capable of traversing the blood–brain barrier [82]. Nanoparticles can also cross the placental barrier, exerting therapeutic effects simultaneously on both the mother and fetus [13]. So far, numerous clinical trials have been conducted on MSC-Exos, with cancer patients confirming their positive effects and absence of observed side effects, indicating the safety and tolerability of exosome therapy [93]. In terms of tumourigenicity and immune rejection, MSC-Exos transplantation outperforms MSCs. Sun et al. conducted a study evaluating the safety of hUCMSC-Exos transplantation and suggested that they were pyrogen-free, causing no side effects on haemolysis, liver and kidney function, or haematological indices. Notably, they do not induce vascular or muscle stimulation or systemic allergic reactions. The results demonstrated the excellent tolerance of hUCMSC-Exos in animal models [94]. Moreover, exosomes, as inert vesicles, reduce the risk of long-term side effects, such as arrhythmias, calcification, and thrombosis [95, 96]. Unlike MSCs, exosomes lack replication capability, thereby avoiding uncontrolled division and greatly reducing the risk of tumour formation during proliferation [97]. Additionally, the surface of MSC-Exos can be modified to engineer them into ligand-bound exosomes, enabling evasion of immune responses by binding to specific cells targeting damaged tissue [98]. Regarding preservation conditions, exosomes exhibit greater stability, ease of storage, and transport in vitro, with the ability to be stored at -20℃ for 6 months without significant changes in biochemical activity [12].

MSC-Exos encounter several challenges as therapeutic agents. One major challenge is the accurate quantification of microRNAs entering and exiting exosomes, given their minute quantities [99]. Currently, three main platforms are used for microRNA quantification: quantitative reverse transcriptase-polymerase chain reaction (RT-PCR), microarrays, and next-generation sequencing techniques. While next-generation sequencing allows unbiased analysis of miRNAs, it does not provide RNA quantification. Microarray analysis and quantitative RT-PCR assays requires predesigned primers, which may overlook previously unidentified microRNAs. Furthermore, although high levels of sphingomyelin have been detected in exosomes, comprehensive liposome analysis and characterization of exosomal lipids is a recent development [100]. It is crucial to eliminate the interference from unknown secretory factors in exosomes as harmful cytokines in MSCs can also be secreted by paracrine cells [101]. Clinical applications demand time-saving, low-cost, and convenient methods. However, current strategies for isolating exosomes present opposite features, including laborious and inefficient processes, limited capacity, and short-term viability, which restrict the clinical application of exosomes. Additionally, unresolved technical issues such as side effects, optimal therapeutic dosage, and administration routes pose significant challenges for exosome therapy [100]. Further experimental studies on MSC-Exos are necessary before extensive clinical trials can be conducted.

## Conclusions

The pathogenesis of preeclampsia remains unclear, and effective treatment methods are currently lacking. MSC-Exos play vital roles in its potential pathogenesis. Further research on specific molecules within MSC-Exos not only enhances our understanding of preeclampsia’s pathogenesis, but also enables the identification of more specific and sensitive biomarkers for its onset. Additionally, it holds promise for the treatment of preeclampsia. However, both basic and clinical research on the mechanism of action of MSC-Exos in preeclampsia are still in its early stages, necessitating further in vivo and in vitro exploration in the future.

## Data Availability

Not applicable.

## Abbreviation

PE: Preeclampsia
MSC-Exos: Mesenchymal stem cell-derived exosomes
MSCs: Mesenchymal stem cells
miRNA: MicroRNA
HUVECs: Human umbilical vein endothelial cells
MHC-II: Major histocompatibility complex II
MHC-I: Major histocompatibility complex I
COVID-19: Corona virus disease 2019
let-7b: Lethal-7b
FOXO1: Forkhead box protein O1
AKT: Protein kinase B
hUCMSC-Exos: Exosomes derived from human umbilical cord mesenchymal stem cells
ERK: Extracelluar signal-regulated kinase
MMP2: Matrixmetalloproteinase2
TIM3: T cell immunoglobulin domain and mucin domain-3
mTORC1: Mammalian target of rapamycin 1
BRD4: Bromodomain-containing protein 4
FSTL3: Recombinant follistatin like protein 3
EZH2: Enhancer of zeste homolog 2
mTOR: Machanistic target of rapamycin
ADMSC-Exos: Adipose tissue mesenchymal stem cell exosomes
BMMSC-Exos: Bone marrow-derived exosomes
VEGF: Vascular endothelial growth factor
Ang-1: Human angiopoietin-1
PIK3R2: Phosphoinositide-3-kinase regulatory subunit 2
PI3K: Phosphatidylinostiol3-kinase
ICAM-1: Intercelluar cell adhesion molecule-1
SPRED-1: Sprouty-related,EVH1 domain 1
Wnt4: Wingless-type MMTV integration site family,member 4
ROS: Reactive oxygen species
ATP: Adenosine-triphosphate
IL: Interleukin
DC: Dendritic cells
tolDC: Tolerant dendritic cells
Treg: Regulatory T cells
TNF-α: Tumour necrosis factor-α
TGF-β: Transforming growth factor β
NOX: NAPDH oxidase
AF-MSC: MSC derived from human amniotic fluid
LPS: Lipopolysaccharides
IFN-γ: Interferonγ
HIF-1α: Hypoxia inducible factor-1α
PDGF: Platelet-derived growth factor
RVG: Rabies virus glycoprotein
DOPE/NHS: Dioleoyl-phosphatidyl ethanolamine N-hydroxy succinimide
IONP: Incubated iron oxide nanoparticles
MF: Magnetic field
ATV: Atorvastatin
PGZ: Pioglitazone
RT-PCR: Quantitative reverse transcriptase-polymerase chain reaction
